# Assessing the sustainability of two independent voucher-based family planning programs in Pakistan: a 24-months post-intervention evaluation

**DOI:** 10.1186/s40834-023-00244-w

**Published:** 2023-08-22

**Authors:** Syed Khurram Azmat, Moazzam Ali, Md. Mizanur Rahman

**Affiliations:** 1 Department of Technical Services, Marie Stopes Society, Karachi, Pakistan; 2 AAPNA-Institute of Public Health, Jinnah Sindh Medical University, Karachi, Pakistan; 3 Department of Reproductive Health and Research, World Health Organization, Geneva, Switzerland; 4 Research Center for Health Policy and Economics, Hitotsubashi University, Tokyo, Japan

## Abstract

**Introduction:**

Family planning vouchers have emerged as a promising approach to improve coverage of underserved groups or underutilized services. The current study was designed to measure the residual/longer-term impact of two-independent FP voucher programs on women’s practices beyond the program’s life program.

**Methods:**

A cross-sectional survey conducted, as part of the two-independent larger mixed-method studies, approximately 24 months after the close-down of Marie Stopes Society and Greenstar Social Marketing family planning voucher intervention programs in Punjab, Pakistan. Following necessary ethics approvals, 338 voucher MSS clients & 324 voucher GSM clients were interviewed using a structured questionnaire at the household level.

**Results:**

Compared with end-line data, a significant decrease in the modern contraceptive uptake in both MSS (90% at endline to current (*or post-endline*) 52%) and GSM (from 84% to current 56%) intervention sites among the voucher clients was noted. Among MSS voucher clients, the highest decline in use was observed in IUCD (54% at endline versus to current 13%); however no change between the surveys was noted among GSM clients. In both projects, following closure of voucher intervention 34% of the discontinued users in MSS and 29% in GSM sites adopted/switched to a new modern contraceptive again. In the post-intervention survey, wealth-based inequality in GSM data depicts more pro-rich utility for modern methods, indicating pro-rich inequality, in contrast, the post-intervention survey in MSS found mixed results such as pro-poor inequality for any method and modern method use.

**Conclusions:**

The prevalence for contraception in two-independent study sites, following closure of voucher intervention remained high than national average. This study provides evidence that family planning vouchers can bring about an enduring positive change in clients’ behaviours in using modern contraceptive methods among poor populations among both intervention models. These results are useful to design family planning programs that will sustain when the donor funding terminates.

**Supplementary Information:**

The online version contains supplementary material available at 10.1186/s40834-023-00244-w.

## Introduction

Sustainability of healthcare interventions is defined as the ability of a program or implementation strategy to maintain individual behavioral change and produce continued benefits or results over a well-defined period of time even after it evolves or adapts from the initial program intervention [1, 2]. Sustained use of program components and activities past their initial funding period and achievement of intended outcomes is vital in ensuring constant and effective health care improvements over time. Evidently, programs that fail to dedicate time and resources towards building sustainability of their interventions, end up wasting the initial investments over unrealized potential [3].

Sustainability of interventions is dependent on the Consultation, Collaboration and Consolidation processes executed throughout intervention implementation [4]. During the consultation process, transparency, client or service provider buy-in and alignment of measures with organizational or community goals are important in sustaining effective programs [5].

The use of contraception has the potential to reduce maternal and child mortality and contribute to poverty reduction and social and economic development, particularly in countries with high fertility and rapid population growth [6, 7]. However, despite global commitments and targeted efforts to increase the use of contraception, high unmet need for family planning continues to be a major challenge in low- and middle-income countries (LMICs) [8], with stark poor-rich disparities in many parts of the world [9].

Globally, close to one in five women continue to have an unmet need for family planning in 2023 [10]. Several approaches have been used to increase the accessibility of quality family planning services [11]. Demand-side financing (DSF) through vouchers, health insurance and conditional cash transfer [12], is one approach that has been used in LMICs to improve maternal and reproductive health services, and the basic premise of which is to transfer the purchasing power to the clients [13]. Among these, family planning vouchers have emerged as a promising approach to improve fertility- and contraceptive services [12]. Vouchers are considered as a tool for improving quality and improving access for poor and underserved communities, including youth and postpartum [14].

Being the fifth most population country, Pakistan is grappling with several challenges, including rapid population and high unmet need for contraception [15, 16]. The current use of modern contraception stands at 26% [16] which remained unchanged over the last 5 years [17–19]. Female sterilisation and condom being the predominantly used methods [16]. A range of demand- and supply-side factors are responsible for low contraceptive use including, but not limited to, sub-standard quality of services [16], women’s perceived socio-cultural unacceptability of FP [20] and health concerns [16].

Over the last one decade, the country embarked on pro-poor approaches – especially voucher coupons—targeting marginalised communities to improve access to and use of quality maternal [21] and reproductive health services [22, 23] with promising results [24] in improving maternal and reproductive health outcomes institutional birth [24] and contraceptive use [25, 26]. Though effective, voucher programs sustainability, both at programmatic levels and maintenance of user behaviours (i.e. contraceptive use) following completion of funded-projects are seen as potential challenges [27].

Responding to the concerns regarding sustainability of voucher programs, a larger study was conducted to generate empirical evidence by examining the residual impact of two voucher programs – implemented by Marie Stopes Society (MSS) [26] in the rural district of Punjab and Greenstar Social Marketing (GSM) [27] in an urban district of Punjab- on contraceptive behaviours of voucher recipients and franchised service providers after 24-months post intervention. Both the voucher programs have shown a substantial increase in the knowledge and practices regarding contraception [28, 29]. To the best of the authors’ knowledge, limited or scarce research has been conducted to assess the long-term residual impact of voucher programs either in the context of family planning, especially after project close-out, or in the post-intervention period [30].

This paper reports on the residual impact of the two-independent FP voucher programs on practices of women pertaining to contraception.

### Description of voucher intervention programs

- Marie Stopes Society (MSS) voucher program: Lack of FP quality and prohibitive cost were the key assumptions behind MSS single-purpose rural-based free voucher program that restricts women’s access to contraceptive use. The voucher intervention was delivered through an established social franchise network of private service providers. Core components of intervention included: a) pre-paid vouchers for free short- and long-term contraceptive services including method uptake, follow-up for side-effect management and removal of FP; b) provision of FP services through a MSS-trained private sector service providers (mid-level: nurse, midwife, lady health visitor; physician: medical doctor); and c) community outreach workers for distribution of vouchers to women who belonged to poorest two wealth quintiles based on screening criteria over a period of 18 months coverage (within the 29 months total project period). Further details can be found elsewhere [28].
- Greenstar Social Marketing (GSM) voucher intervention program: The premise for GSM multi-purpose urban-based subsidised voucher was lack of awareness among women owing to limited or lack of counselling by service providers. The intervention was to enhance client-provider interaction for improved FP counselling through vouchers. Core components of intervention included: a) booklet of vouchers that offer 13 pre-paid visits over a period of 18 months coverage (within the 31 months total project period) that comprised of two postnatal care visits, five infant immunisation and six FP visits (including contraceptive); b) cost of the voucher booklet was 50 Pakistan rupee (0.27 cents – USD 1 = 183 PKR); c) vouchers were distributed through service providers and distributing agencies to women belonging to poorest two wealth quintile as per screening criteria. Further details can be found elsewhere [29].

## Methods

### Study design

This paper reports on a cross-sectional survey conducted, as part of the larger mixed-method study, approximately 24-month after the close-down of MSS and GSM contraceptive voucher programs.

### Study settings

The survey was conducted during May–June 2017 in the same geographic catchments in districts of Chakwal and Faisalabad where MSS and GSM voucher interventions were implemented, respectively. MSS engaged a total of 25 social franchise providers in the intervention district; out of those, 12 providers were randomly selected for the intervention group. For the current study, six service provider sites were randomly selected. In contrast, GSM implemented its voucher program through 100 social franchise providers in the intervention districts, but 25 service providers were randomly selected for the intervention group. For the current study, sixteen providers were randomly selected for this survey.

### Study population

Women of reproductive age (15–49 years) who had received a voucher (booklet) during intervention period were eligible to participate in the survey.

### Sampling

We went through multiple steps from the development of list frame of voucher clients to the actual interviews. The same sampling strategy was used for both the programs. At first, we developed a list frame of all voucher clients served through the programs (MSS = 7,101 and GSM = 28,000). Second, we randomly selected 12 MSS and 25 GSM service providers. However, of the selected service providers, 6 MSS and 9 GSM couldn’t participate in the survey due to non-availability/shifted/migrated (MSS = 4 and GSM = 5), non-cooperation (MSS = 2 and GSM = 2), and death (GSM = 2). Consequently, the list frame of voucher clients for the remaining active providers reduced to 638 in MSS and 567 in GSM. Finally, the desired sample of 400 participants from each programs was conveniently recruited from the list frame.

### Data collection tools

A standardized structured questionnaire was developed for both the MSS and GSM survey. The questionnaire broadly covered: a) socio-economic and demographic characteristics; b) reproductive history; c) fertility preferences and awareness and use of contraception; d) method discontinuation and its reasons; and e) experiences regarding use of voucher-based contraceptive services. The questionnaire was translated into Urdu and revised based on pre-testing conducted among 36 (18 each MSS and GSM) married women of reproductive age. These were not included in the final sample.

### Data collection and management

Face-to-face interviews were conducted by trained data collectors (all female) on paper-based structured questionnaire at participants’ home in privacy. On average, each interview took 20–30 min. We took several measures to ensure the quality of collected data. In the field, all the forms were checked routinely for completeness and logical errors. Principal Investigators also conducted monitoring visits to ensure that data were collected in adherence with the study protocol. Data were double-entered on a pre-designed data entry software that was developed in Epidata version 3.1. The software was also restricted for the must-filled entries and extreme values.

### Statistical analysis

Data analysis was performed separately for MSS and GSM with no intention to compare the two independent voucher programs. Simple frequencies and percentages were used to explore the socio-demographic and reproductive health characteristics of study participants. The residual impact of the voucher programs was assessed by comparing the estimates of current survey with the respective endline surveys of each programs conducted back in 2015. Crude differences between current survey and the endline were tested using Pearson chi-square (for categorical variables) and t-test (for continuous variables). Finally, we applied multivariable logistic regression to observe changes in the use of contraceptive methods between endline (2015) and current survey, after adjusting for potential covariates such as age, number of children, education status and wealth quintile. A *p*-value of ≤ 0.05 was taken to indicate statistical significance.

For the purpose of health equity analysis, the index for the wealth quintile was constructed according to the technique used internationally by the Demographic and Health Survey (DHS) [29]. Households were divided into five wealth quintiles (Q1-Q5) to assess socio-economic status. Slope index of inequality (SII) and relative index of inequality (RII) were calculated to measure absolute and relative measure of inequality, respectively. SII measures the absolute difference between the extreme wealth quintiles (Q5-Q1) and reflects the difference in percentage points in a given family planning indicators, while RII is a measure of ratio and expressed by percentage. Both SII and RII were calculated using linear regression models. Additionally, a concentration curve was constructed to illustrate the socio-economic inequality in the indicators graphically and a concentration index was calculated to measure the extent of socio-economic inequality in each family planning indicators. All statistical analyses were performed using Stata version 14.1/MP (StataCorp, College Station, Texas USA).

### Ethical consideration

The study protocol was approved by the World Health Organization ‘Scientific and Ethics Review Committee (Project ID: A65911). All enumerators were trained to ensure that they took appropriate measures in order protect the confidentiality of participants to the fullest extent possible. All study participants gave written, voluntary informed consent.

#### Patient and public involvement

No patients were involved in this research. The voucher-based contraceptive users’ participants were recruited not as patients, but as service users and as citizens. Hence, the development of research questions and outcome measures was not informed by the patients’ priorities, experiences, and preferences. Patients were not involved in the design of the research. Further, this is not clinical research or randomized clinical trial.

## Results

At MSS study sites, a total of 638 clients were approached, 338 (53.0%) were successfully interviewed. The main reasons for non-response were: household could not be located (225, 35.3%), not at home (47, 15.8%) and migrated (26,8.7%). Similar response rate was found at GSM sites where (324, 57.1%) participants were successfully interviewed of 567 who were approached. The main reasons for non-response were: household not located (119, 21.0%), not at home (34,6.0%), and a few migrated (10, 1.7%).

Table 1 compares the characteristics of study participants (who had used vouchers), between endline and post-endline survey across MSS and GSM voucher programs. With few exceptions, the characteristics of participants differed between the endline and ‘post-endline’ survey across both the programs. However, it is to note that across both MSS and GSM sites, the actual differences were substantive for characteristics such as age, age at marriage, mean number of children and mean household members (see Table 1).

**Table 1:** Comparison of participants’ characteristics between endline and post-endline survey, according to MSS and GSM voucher programmes

**Characteristics**	**MSS – single purpose voucher**					**GSM – multipurpose voucher**				
	**Endline *n* = 390**		**Post-endline *n* = 338**		***p*-value**	**Endline *n* = 409**		**Post-endline *n* = 324**		***p*-value**
	**n**	**%**	**n**	**%**		**n**	**%**	**n**	**%**	
Women age					< 0.001					< 0.001
Mean (SD)	31.2	(5.4)	32.6	(5.4)		29.3	(± 5.0)	32.0	(± 5.1)	
Age of women at marriage					< 0.001					< 0.001
Mean (SD)	19.7	(3.2)	20.2	(3.0)		21.1	(± 3.5)	19.6	(± 2.4)	
Women education					< 0.001					0.03
None/no formal	71	18.2	72	21.6		138	33.7	97	30.5	
Completed primary/some secondary	184	47.2	110	32.9		185	45.2	127	39.9	
Completed secondary or above	135	34.6	152	45.5		86	21.0	94	29.6	
Number of children					< 0.001					< 0.001
Mean (SD)	2.9	(1.4)	3.1	(4.1)		2.6	(± 1.4)	3.1	(± 1.5)	
Women’s employment					0.003					0.943
Housewife	378	96.9	311	92.0		396	96.8	314	96.9	
Working women	12	3.1	27	8.0		13	3.2	10	3.1	
Household members					< 0.001					< 0.001
Mean (SD)	5.2	(± 1.6)	6.2	(± 2.1)		6.6	(± 2.6)	5.9	(± 2.1)	
Wealth quintile					0.753					< 0.001
Poorest	171	21.6	133	20.1		39	9.7	65	20.1	
Poor	152	19.2	133	20.1		35	8.7	65	20.1	
Medium	156	19.7	132	19.9		75	18.6	65	20.1	
Wealthy	174	22.0	134	20.2		125	31.0	66	20.4	
Wealthiest	139	17.6	130	19.6		129	32.0	63	19.4	

*MSS* Marie Stopes Society, *GSM* Greenstar Social Marketing

Table 2 presents the comparison of current contraceptive use among voucher recipients between endline and post-endline survey. At MSS sites, of the voucher clients interviewed at endline survey, 90.8% reported to be using any form of contraceptive method, which has significantly reduced to 58.3% at post-endline survey. This decrease was observed primarily in the use of modern methods (from 90.3% to 51.8%); interestingly, the use of traditional methods increased from 0.8% in the endline to 5.3% in the post-endline survey. Among modern methods, the highest decline was recorded in the use of IUCD (from 53.6% in the endline study to 13.0% in post-endline), followed by implants (from 7.0% to 3.0%); whereas, the use of pill, injectable and condom remained more or less the same. Interestingly, a significant increase was noted in the use of tubal ligation (from 0.8% to 5.3%).

**Table 2:** Comparison of current contraceptive use between endline and post-endline survey, according to MSS and GSM voucher programmes

**Characteristics**	**MSS – single purpose voucher**					**GSM – multipurpose voucher**				
	**Endline *n* = 390**		**Post-endline *n* = 338**		***p*-value**	**Endline *n* = 409**		**Post-endline *n* = 324**		***p*-value**
	**n**	**%**	**n**	**%**		**n**	**%**	**n**	**%**	
Any method	354	90.8	197	58.3	< 0.001	356	87.0	250	77.2	< 0.001
Any modern method	352	90.3	175	51.8	< 0.001	343	83.9	180	55.6	< 0.001
Oral pill	20	5.1	15	4.4	0.664	33	8.1	11	3.4	0.008
IUCD	209	53.6	44	13.0	< 0.001	126	30.8	100	30.9	0.987
Injectable	40	10.3	38	11.2	0.668	92	22.5	17	5.2	< 0.001
Implant	29	7.4	10	3.0	0.007	1	0.2	0	0.0	N/A
Condom	51	13.1	53	15.7	0.317	80	19.6	36	11.1	0.002
Tubal ligation	3	0.8	18	5.3	< 0.001	12	2.9	16	4.9	0.160
Any traditional method	2	0.5	19	5.6	< 0.001	12	2.9	70	21.6	< 0.001
Periodic abstinence	0	0.0	3	0.9	0.062	2	0.5	27	8.3	< 0.001
Lactational amenorrhea	2	0.5	8	2.4	0.032	0	0.0	7	2.2	0.003
Withdrawal	0	0.0	8	2.4	0.002	10	2.4	36	11.1	< 0.001

In contrast, the drop in the proportion of any contraceptive method from endline (87%) to post-endline (77.2%) was comparatively low at GSM sites. This decrease was observed primarily in the use of modern methods (from 83.9% to 55.6%); interestingly, we observed a substantive increase in the use of traditional methods from 2.9% in the endline survey to 21.6% in the post-endline survey. Among modern methods, the highest decline was recorded in the use of injectables (from 22.5% in the endline survey to 5.2% in the post-endline survey), followed by condom (from 19.6% to 11.1%) and pill (from 8.1% to 3.4%).

According to the voucher clients at MSS sites in Table 3, the information oncontraceptive methods received was universal at endline; however, among current users at post-endline, around equal proportion (14%) of the women reported that they were not informed about potential side effects of the method or what to do if they experienced side effects. On the other hand, the GSM clients (Table 3) reported receiving information on contraceptive methods increased significantly from endline to post-endline survey across all three indicators at GSM sites. Although, the information related to the contraceptive methods received remained high at MSS and GSM service providers compared to the endline (see Supplementary Table 1).

**Table 3:** Clients’ experiences of quality of FP services

**Characteristics**	**MSS – single purpose voucher**					**GSM – multipurpose voucher**				
	**Endline *n* = 351**		**Post-endline *n* = 171**		***p*-value**	**Endline *n* = 309**		**Post-endline *n* = 179**		***p*-value**
	**n**	**%**	**n**	**%**		**n**	**%**	**n**	**%**	
Informed about side effect or problems of method use	351	100.0	148	86.5	< 0.001	268	86.7	177	98.9	< 0.001
Informed what to do in case of side effects	350	99.7	148	86.5	< 0.001	261	84.5	176	98.3	< 0.001
Informed about range of contraceptive methods	351	100.0	167	97.7	0.004	286	92.6	178	99.4	< 0.001

As reported in Table 4, post-endline survey revealed that 85% and 77.2% of the voucher clients of MSS and GSM reported to have discontinued the method they had adopted through voucher two years ago during the intervention period, respectively. Reportedly, IUD was the most commonly cited method that was last discontinued in the 24 months across MSS (44.6%) and GSM (52.1%) clients. Pregnancy desire (MSS = 29.1%, GSM (25.2%) and side effects/fear of health concerns (MSS = 22.4%, GSM = 40.5%) constituted the most commonly cited reasons for discontinuation among MSS and GSM clients, which also aligns to the reasons reported in the most recent demographic survey.

**Table 4:** Contraceptive discontinuation within last 2 years (following the closure of voucher programme) and reasons for discontinuation, according to MSS and GSM voucher programmes

	**MSS – single purpose voucher**		**GSM – multipurpose voucher**	
	**Post-endline *n* = 338**		**Post-endline *n* = 324**	
	**n**	**%**	**n**	**%**
Clients discontinued the method adopted through voucher	275	85.0	250	77.2
**Method discontinued within last 24 months**	**(*n* = 165)**		**(*n* = 234)**	
Oral pill	15	9.0	45	19.2
IUD	74	44.6	122	52.1
Injectable	20	12.0	31	13.2
Implant	29	17.5	0	0.0
Condom	27	16.3	36	15.4
**Reasons for discontinuation**	**(*n* = 215)**		**(*n* = 369)**	
Pregnancy desire	65	29.1	97	25.2
Side effect/Fear of health concerns	50	22.4	156	40.5
Others (accessibility, inconvenient, God’s Will, Husband out of country)	32	17.8	49	16.9
Infrequent sex	33	14.8	14	3.6
Hysterectomy	13	5.8	15	3.9
Husband opposition	11	4.9	5	1.3
Method failure	11	4.9	33	8.6

Table 5 presents wealth-based inequalities in access to different contraceptive use by endline and post-endline survey for MSS and GSM voucher programs separately. At MSS sites, around 90% of the poor population used modern methods in the endline survey, while this declined to around 53% in the post-endline survey. Use of all contraceptive methods was concentrated among poorer families in the endline survey, however at post-endline survey there was pro-poor inequality for any method and modern method. The slope index of inequality indicated that in the post-endline survey, use of modern methods among the rich population was around 9 percentage points lower than among the poor population. The relative index of inequality indicates that the richest respondents were 0.8 times less likely to use modern contraceptives compared to the poorest quintile group. Similarly, the concentration index identified that the use of modern contraceptives was concentrated among the poor group in both surveys.

**Table 5:** Wealth-based inequalities in contraceptive use in endline and post-endline survey, according to MSS and GSM voucher programmes

	**Coverage, (%)**		**Inequality assessment**		**Concentration index (x100)**
	**Q1 (Poorest)**	**Q5 (Richest)**	**SII (Q5: Q1, % points)**	**RII (Q5:Q1)**	
**MSS – single purpose voucher**					
**Endline (2015)**					
Modern method user	90.2 (83.7-94.2)	100	0.9 (-9.0 to 10.9)	1.0 (0.9 to 1.1)	-2.4 (-4.2 to -0.5)
Traditional use	1.5 (0.38-5.9)	0	-2.3 (-5.8 to 1.1)	0.2 (-0.1 to 0.6)	-40.6 (-121.2 to 40.0)
**Post-endline (2017)**					
Modern method user	52.9 (41.0-64.6)	46.3 (34.6-58.3)	-9.4 (-27.8 to 0.1)	0.8 (0.5 to 1.1)	-10.0 (-15.8 to -4.2)
Traditional use	NA	NA	4.5 (-4.2 to 13.1)	1.9 (-0.5 to 4.4)	15.3 (-10.0 to 40.6)
**GSM – multipurpose voucher**					
**Endline (2015)**					
Modern method user	97.4 (83.5-99.7)	79.8 (72.0-85.9)	-15.7 (-27.2 to -4.1)	0.8 (0.7 to 0.9)	-6.3 (-8.7 to -3.9)
Traditional use	0.00	2.3 (0.8-7.0)	-0.8 (-5.5 to 4.2)	0.8 (-0.2 to 1.9)	10.4 (-22.5 to 43.3)
**Post-endline (2017)**					
Modern method user	53.8 (41.6-65.6)	58.7 (46.1-70.3)	10.4 (-8.3 to 29.0)	1.2 (0.8 to 1.6)	-3.6 (-9.3 to -2.0)
Traditional use	23.0 (14.3-35.0)	20.6 (12.3-32.5)	-5.6 (-21.1 to 9.9)	0.8 (0.2 to 1.3)	-0.3 (-12.4 to 11.8)

*SII* Slope index of inequality, *RII* Relative index inequality, *95% CI* 95% Confidence interval

All equity analysis was adjusted for base and endline survey points

The endline survey curve indicated that there is no inequality to regarding the use of modern contraceptive methods among poor and rich, however, greater pro-poor inequality was observed in the post-endline survey (see Supplementary Fig. 1).

With respect of GSM sites, the use of modern contraceptive methods in the endline survey was higher among the disadvantaged group than the affluent group, but the use of modern contraceptives declined among both groups in the post-endline survey. Pro-poor inequality in the endline survey and pro-rich inequalities were observed for all types of contraceptive user group. The slope index of inequality indicated that the use of modern contraceptive method was about 15.7-percentage points lower in the richest group in the endline survey than the poorest quintile group. However, in the post-endline survey, the use of modern contraceptives was 10.4 percentage points higher in the richest quintile group than the poorest quintile group. the concentration curve for the use of modern contraceptive in the endline survey was above the line of equality, indicating that modern contraceptives use was more concentrated among the poorest group in the endline survey. In post-endline survey, the concentration curve for modern contraceptive moved far below the line of inequality, indicating pro-rich inequality (see Supplementary Fig. 2).

## Discussion

Attention to the sustainability of health intervention programs is increasing, a development program was considered sustainable when it is able to deliver an appropriate level of benefits for an extended period of time after major financial, managerial and technical assistance from an external donor is terminated. Less research has been conducted to determine what happens beyond that point [31].

A programme that is considered well designed to affect health status is vulnerable, particularly to withdrawal of funding, if it has neglected the importance of stakeholders driving or hindering the programme. The stakeholders including the public sector need to be engaged in the program design and implementation strategy, and the roles and responsibilities should then be clarified. The engagement plays a vital role in sustained achievement of desirable program goals and population outcomes. Irrespective of the type of health interventions (such as vouchers), the key to sustainability is how the stakeholders identify, define, prioritize, and own the health issues.

The data from our study has shown that positive behaviour persists even after the intervention ends, so following investment in the infrastructure in establishing the intervention in health care, the donors need to develop a clear pathway on how over the period of implementation the program, ownership will be gradually handed over to the key stakeholders in a stepwise manner before the fundings end. Thus, a well-thought-out plan can help sustain the desired changes beyond the time of the project intervention and can help to institutionalized it.

There is dearth of evidence to questions like, what happens to the short-term projects funded by government agencies and foundations to foster local improvement in some aspect of health or health promotion? This paper attempts to address the issues raised here.

Family planning vouchers have emerged as a promising approach to improving fertility outcomes [14]. However, no research has attempted to measure the sustainable impact of voucher programs either in the context of maternal health or family planning.

After the closure of voucher intervention for 24 months a considerable decrease was noted in the use of modern contraceptive methods in both MSS (38.5%) and GSM (28.3%) study sites. However, this decrease was still higher than the national average of modern method use, highlighting the positive behaviour change in contraceptive use in the two independent voucher interventions. Previous research from a similar social franchising context in Pakistan showed higher continuation [32–34] compared with national statistics [16]. As the voucher intervention closed, a substantial rise in traditional methods was observed in both the projects – particularly among GSM clients (6% points in MSS and 18% points in GSM). This perhaps signifies an increase in awareness and subsequently the demand but few possible factors such as affordability, lack of proper supervision from field health worker can be cited. However, further research is needed to understand the increase in the use of traditional methods following end of intervention.

Despite that huge reduction in the use of modern contraceptive from endline to post-endline in both the projects, however, it was highly encouraging to observe that 34% of the relapsed users in MSS and 29% in GSM sites adopted a modern contraceptive again. This switching proportion is comparatively far higher than the national statistics of 3.4% [16]. Notably, approximately two years after the closure of the voucher program, the current use of modern contraceptive in both the study sites was higher than the national contraceptive use [16]. Moreover, the use of modern methods at post-endline survey in the MSS project was similar to the proportion observed at endline in the general population (MSS = 50%) [28]; whereas in the case of GSM, the post-endline modern contraceptive use was far higher than the endline proportion (GSM = 32%) [29]. These results are indicative of an enduring change in the behaviour of communities that occurred as a result of the voucher intervention program.

Among MSS voucher clients, the highest decline was observed in IUCD use. In GSM sites, a considerable decrease was observed in the use of short-acting methods. This discontinuation could have been prevented through engagement of LHWs who are responsible for door-to-door visits for counselling and provision of short-acting methods. It is therefore imperative that for any new initiative, programmatic synergies should be explored with existing public health system, and where possible, health system linkages should be established accordingly.

Among those exposed to voucher program, about half of the contraceptive users in GSM and one-third in MSS adopted the new method from service providers and 25% of the respondent cited MSS/GSM project field worker as the source of the referrals. These findings not only reinforce the importance of demand generation for any future intervention through outreach workers such as Lady Health Workers, FWWs, social mobilizers, CMWs, vaccinators etc.; but at the same time it signifies provision of quality services that establishes a long-term relationship between service providers and the clients.

With respect to service quality, in MSS we observed a significant decrease of clients being informed about potential side effects and follow up comparing endline to post-endline survey. This could be that the quality of service provision got comprised in the absence of clinical supervision and monitoring as it was during voucher intervention. On the contrary, a significant increase was observed in the GSM site where provision of this information was nearly universal, irrespective of the source of method. Overall, these results are still better than national estimates where only one-third of the clients are provided with such information [16]. This increase in informed choice may be attributed to the fact that service providers have actually realised the importance of quality counselling and hence have been able to maintain the standard.

Health equity analysis showed mixed results for MSS and GSM voucher clients. In GSM at the endline survey, the use of modern contraceptives was higher in the poorer group compared to the richest, which reversed in the post-endline survey where use was higher in the richest group. These findings reinforce the importance of the continuity of a voucher or similar type of intervention which encourages the poorest quintile group access to the use of modern contraceptive methods. On the other hand, in MSS study sites, the use was higher in poorer segments of the population than in the wealthiest segments at post-endline. A possible explanation could be that MSS service providers were primarily based in rural areas it is common practice to maintain good rapport with the clients. In addition, the use of free voucher may have changed the behaviour of users in the intervention catchment suggesting ex-voucher user either continuing to use the same method or adopted a new method by paying out-of-pocket. However, this merits further investigation for better understanding.

Side effects were cited as the most common reasons for discontinuation among GSM clients (41%) whereas this was the second most common reason among MSS clients (22%). In both the projects, side effects as the reason for discontinuation increased from endline to post-endline survey, highlighting lack of supportive structure that was there during intervention. Moreover, high IUCD expulsion rate (9% in GSM and 5% in MSS) was also documented in our study. These findings suggest the need for better counselling of clients, follow-up support for side effect management [35] and enhancement of provider skills (on IUCD) in procedures. Keeping in view of women’s restricted mobility in Pakistan, technological solutions could be considered in the communities such as helpline services where women could call and get information regarding side effect management [35].

### Limitations

There are some limitations in our study. There is possibility of recall problem in answering questions about their past experiences with service providers such as information of method at the time of uptake, last method discontinued etc.; matched pre-post design whereby interviewing same voucher clients would have accounted for the differences in sample characteristics and other unobserved confounders. This was minimized as information on personal identifiers was discarded by the implementing organisations (MSS and GSM); locating households with the support on field teams who are involved in the distribution of vouchers may have introduced a selection bias as they could recall and take data collectors to those household where they have good relationship with women – and this may have skewed the responses positively in favour of the intervention; finally, as contraceptive calendar approach was not used, the study is unable to capture and report exact time of method discontinuation.

## Conclusion

Understanding sustainability of interventions is important in forming a baseline for promoting effective practices that yield desired health outcomes over longer period [35].

Present evidence suggests that vouchers can contribute to the agenda of equitable access in universal health coverage by addressing its three core aspects: 1) Increase access to the poor and vulnerable; 2) Increase the number of services by ensuring the availability of contraceptives in line with the national policies, and 3) provides financial protection by being inclusive and reaching out to the underserved. This study also indicates that vouchers can bring about an enduring positive change in behaviours regarding the use of modern contraceptive methods among poor populations using the MSS led single-purpose free rural voucher program. However, method discontinuation as a result of side effects seems to be a common practice that should be dealt with properly through improved counselling and active follow-up. This study’s findings will inform policymakers to plan services to support and sustain positive health behaviour among the population when donor funding goes dry.

### Supplementary Information

**Additional file 1: Supplementary Table 1.** Clients’ perception regarding quality of FP services at post-endline according to type of service provider at MSS and GSM voucher programmes.

**Additional file 2: Supplementary Figure 1.** Concentration curve of modern contraceptive use across endline and post-endline survey MSS voucher programme.

**Additional file 3: Supplementary Figure 2.** Concentration curve of modern contraceptive use across endline and post-endline survey GSM voucher programme.

## References

[1] Moore JE, Mascarenhas A, Bain J, Straus SE (2017). Developing a comprehensive definition of sustainability. Implement Sci.

[2] Walugembe DR, Sibbald S, Le Ber MJ, Kothari A (2019). Sustainability of public health interventions: where are the gaps?. Heal Res Policy Syst.

[3] Herkama S, Kontio M, Sainio M, Turunen T, Poskiparta E, Salmivalli C (2022). Facilitators and barriers to the sustainability of a school-based bullying prevention program. Prev Sci.

[4] Khalil K, Kynoch K (2021). Implementation of sustainable complex interventions in health care services: the triple C model. BMC Health Serv Res.

[5] Mendelson A (2017). The effects of pay-for-performance programs on health, health care use, and processes of care: a systematic review. Ann Intern Med.

[6] Cleland J, Bernstein S, Ezeh A, Faundes A, Glasier A, Innis J (2006). Family planning: the unfinished agenda. Lancet.

[7] Ahmed S, Li Q, Liu L, Tsui AO (2012). Maternal deaths averted by contraceptive use: an analysis of 172 countries. Lancet.

[8] Kantorova V, Wheldon MC, Ueffing P, Dasgupta ANZ (2020). Estimating progress towards meeting women’s contraceptive needs in 185 countries: a Bayesian hierarchical modelling study. PLoS Med.

[9] Ross J (2015). Improved reproductive health equity between the poor and the rich: an analysis of trends in 46 low- and middle-income countries. Glob Health Sci Pract.

[10] United Nations Population Division: www.population.un.org/dataportal/home. Accessed on 16 Apr 2023.

[11] Mwaikambo L, Speizer IS, Schurmann A, Morgan G, Fikree F (2011). What works in family planning interventions: a systematic review. Stud Fam Plann.

[12] Bellows B, Bulaya C, Inambwae S, Lissner CL, Ali M, Bajracharya A (2016). Family planning vouchers in low and middle income countries: a systematic review. Stud Fam Plann.

[13] Murray S, Hunter B, Bisht R, Ensor T, Bick D (2014). Effects of demand-side financing on utilisation, experiences and outcomes of maternity care in low- and middle-income countries: a systematic review. BMC Pregnancy Childbirth.

[14] High Impact Practices in Family Planning (HIP). Family planning vouchers: a tool to boost contraceptive method access and choice. Washington, DC: HIPs partnership; 2020. Available from: https://www.fphighimpactpractices.org/briefs/familyplanning-vouchers.

[15] United Nations Population Fund (UNFPA) (2013). Pathfinder international. The state of family planning in Pakistan: targeting the missing links to achieve development goals.

[16] Khan A, Shaikh BT (2013). An all time low utilization of intrauterine contraceptive device as a birth spacing method- a qualitative descriptive study in district Rawalpindi, Pakistan. Reprod Health.

[17] National Institute of Population Studies (NIPS) Pakistan. Pakistan Demographic and Health Survey 2006-07. Islamabad, Pakistan and Columbia, Maryland, USA: NIPS and IRD/Macro International Inc; 2008.

[18] National Institute of Population Studies (NIPS) Pakistan and ICF. Pakistan Demographic and Health Survey. 2012-13. Islamabad, Pakistan, and Calverton, Maryland, USA: NIPS and ICF; 2013.

[19] National Institute of Population Studies (NIPS) Pakistan and ICF. Pakistan Demographic and Health Survey 2017-18. Islamabad, Pakistan, and Rockville, Maryland, USA: NIPS and ICF; 2019.

[20] Casterline JB, Sathar ZA, Haque MU (2001). Obstacles to contraceptive use in Pakistan: a study in Punjab. Stud Fam Plann.

[21] Agha S (2011). Changes in the proportion of facility-based deliveries and related maternal health services among the poor in rural Jhang, Pakistan: results from a demand-side financing intervention. Int J Equity Health.

[22] Azmat SK, Shaikh BT, Hameed W, Mustafa G, Hussain W, Asghar J (2013). Impact of social franchising on contraceptive use when complemented by vouchers: a quasi-experimental study in Rural Pakistan. PLoS One.

[23] Azmat SK, Hameed W, Hamza HB, Mustafa G, Ishaque M, Abbas G (2016). Engaging with community-based public and private mid-level providers for promoting the use of modern contraceptive methods in rural Pakistan: results from two innovative birth spacing interventions. Reprod Health.

[24] Mehboob G, Shaikh BT (2015). Experience of vouchers for reproductive health services in developing countries: making a case for Pakistan through a systematic review. J Ayub Med Coll Abbottabad.

[25] Broughton EI, Hameed W, Gul X, Sarfraz S, Baig IY, Villanueva M (2017). Cost-effectiveness of a family planning voucher program in Rural Pakistan. Front Public Health.

[26] Ali M, Azmat SK, Hamza HB, Rahman MM, Hameed W (2019). Are family planning vouchers effective in increasing use, improving equity and reaching the underserved? An evaluation of a voucher program in Pakistan. BMC Health Serv Res.

[27] Ali M, Azmat SK, Hamza HB, Rahman MM (2020). Assessing Effectiveness of multipurpose voucher scheme to enhance contraceptive choices, equity, and child immunization coverage: results of an interventional study from Pakistan. J Multidiscip Healthc.

[28] Azmat SK, Ali M, Hameed W, Mustafa G, Abbas G, Ishaque M, Bilgrami M, Temmerman M (2014). A study protocol: using demand-side financing to meet the birth spacing needs of the underserved in Punjab Province in Pakistan. Reprod Health.

[29] Rutstein SO, Johnson K (2004). DHS comparative reports 6: the DHS wealth index.

[30] Ali M, Azmat SK, Hamza HB (2018). Assessment of modern contraceptives continuation, switching and discontinuation among clients in Pakistan: study protocol of 24-months post family planning voucher intervention follow up. BMC Health Serv Res.

[31] US Agency for International Development (1988). Sustainability of development programs: a compendium of donor experience.

[32] Hameed W, Azmat SK, Ali M, Ishaque M, Abbas G, Munroe E (2016). Comparing effectiveness of active and passive client follow-up approaches in sustaining the continued use of Long Acting Reversible Contraceptives (LARC) in Rural Punjab: a multicentre, non-inferiority trial. PLoS One.

[33] Azmat S, Shaikh B, Hameed W, Bilgrami M, Mustafa G, Ali M (2012). Rates of IUCD discontinuation and its associated factors among the clients of a social franchising network in Pakistan. BMC Womens Health.

[34] Hameed W, Azmat S, Ishaque M, Hussain W, Munroe E, Mustafa G (2015). Continuation rates and reasons for discontinuation of intra-uterine device in three provinces of Pakistan: results of a 24-month prospective client follow-up. Health Res Policy Syst.

[35] Stirman SW, Kimberly J, Cook N, Calloway A, Castro F, Charns M (2012). The sustainability of new programs and innovations: a review of the empirical literature and recommendations for future research. Implement Sci.

