# Supplementary material for: Assessing the sustainability of two independent voucher-based family planning programs in Pakistan: a 24-months post-intervention evaluation
Source: Contracept Reprod Med. 2023 Aug 22;8:43. doi: 10.1186/s40834-023-00244-w (PMC10464259; doi:10.1186/s40834-023-00244-w)
Supplement: Supplementary file 1 — Additional file 1: Supplementary Table 1. Clients’ perception regarding quality of FP services at post-endline according to type of service provider at MSS and GSM voucher programmes. [file 40834_2023_244_MOESM1_ESM.docx]

**Supplementary Table 1: Clients’ perception regarding quality of FP services at post-endline according to type of service provider at MSS and GSM voucher programmes**

| **Characteristics** | **Informed choice** | | | | | |
| --- | --- | --- | --- | --- | --- | --- |
|  | **Informed about side effects** | | **Informed what to do in case of side effects** | | **Informed about range of methods** | |
|  | **n** | **%** | **n** | **%** | **n** | **%** |
| **MSS voucher programme** | **(n=148)** | | **(n=148)** | | **(n=167)** | |
| Suraj centres | 50 | 98.0 | 51 | 100.0 | 51 | 100.0 |
| Lady Health Workers | 39 | 79.6 | 38 | 77.6 | 48 | 98.0 |
| Govt. Hospital/ RHSC | 40 | 87.9 | 40 | 90.9 | 44 | 99.0 |
| Other private sources | 19 | 75.6 | 19 | 75.6 | 24 | 91.1 |
|  |  |  |  |  |  |  |
| **GSM voucher programme** | **(n=174)** | | **(n=173)** | | **(n=175)** | |
| Greenstar Providers | 85 | 100.0 | 85 | 100.0 | 84 | 98.8 |
| Lady Health Workers | 16 | 94.1 | 16 | 94.1 | 17 | 100.0 |
| Govt. Hospital/ RHSC | 31 | 100.0 | 30 | 95.2 | 31 | 100.0 |
| Other private sources | 42 | 99.2 | 42 | 97.6 | 43 | 100.0 |
