# Supplementary material for: Assessing the sustainability of two independent voucher-based family planning programs in Pakistan: a 24-months post-intervention evaluation
Source: Contracept Reprod Med. 2023 Aug 22;8:43. doi: 10.1186/s40834-023-00244-w (PMC10464259; doi:10.1186/s40834-023-00244-w)
Supplement: Supplementary file 2 — Additional file 2: Supplementary Figure 1. Concentration curve of modern contraceptive use across endline and post-endline survey MSS voucher programme. [file 40834_2023_244_MOESM2_ESM.docx]

**Supplementary Figure 1: Concentration curve of modern contraceptive use across endline and post-endline survey MSS voucher programme**

Modern contraceptive use

Cumulative Proportion of Population
