# Supplementary material for: Assessing the sustainability of two independent voucher-based family planning programs in Pakistan: a 24-months post-intervention evaluation
Source: Contracept Reprod Med. 2023 Aug 22;8:43. doi: 10.1186/s40834-023-00244-w (PMC10464259; doi:10.1186/s40834-023-00244-w)
Supplement: Supplementary file 3 — Additional file 3: Supplementary Figure 2. Concentration curve of modern contraceptive use across endline and post-endline survey GSM voucher programme. [file 40834_2023_244_MOESM3_ESM.docx]

**Supplementary Figure 2: Concentration curve of modern contraceptive use across endline and post-endline survey GSM voucher programme**

Modern contraceptive use

Cumulative Proportion of Population
